# Compressive Strength and Durability of FGD Gypsum-Based Mortars Blended with Ground Granulated Blast Furnace Slag

**DOI:** 10.3390/ma13153383

**Published:** 2020-07-30

**Authors:** Min Pang, Zhenping Sun, Huihao Huang

**Affiliations:** 1 School of Materials Science and Engineering, Tongji University, Shanghai 201804, China; pangmin@tongji.edu.cn; 2 Key Laboratory of Advanced Civil Engineering Materials of Ministry of Education, Tongji University, Shanghai 201804, China; 3 Shanghai Urban Construction Material Co., Ltd., Shanghai 200063, China; huanghuihao_tj@126.com

**Keywords:** flue gas desulfurization (FGD) gypsum, compressive strength, granulated blast furnace slag, ettringite, steam curing condition

## Abstract

One new flue gas desulfurization (FGD) gypsum-based binder is attempted in this article, which is made up of FGD gypsum, ground granulated blast furnace slag (GGBS) and ordinary Portland cement (OPC). Influences of raw materials, chemical activators, and curing conditions on the compressive strength of this new binder-based mortar, as well as its durability performances and microscopic characteristics, are investigated in consideration of utilizing FGD gypsum as much as possible. Results show that the compressive strength of this new binder-based mortar under normal curing conditions could increase along with GGBS dosages from three days to 90 days. The compressive strength of one selected mix proportion (FG-4550), which contains the highest dosage of FGD gypsum (45 wt.%), is much the same as those containing the highest dosage of GGBS. A better compressive strength of FG-4550 under normal curing conditions could be gained if the fineness of GGBS is improved. The activated effect of CaCl_2_ on the compressive strength of FG-4550 is superior to that of Ca(OH)_2_ under steam curing conditions. FG-4550 shows a good capacity for resistance to water, a low shrinkage ratio, but poor compressive strength after 30 freeze-thaw cycles. Based on the mineralogy of X-ray diffraction, the morphology of scanning electron microscopy and the pore diameter distributions of ^1^H nuclear magnetic resonance, the compressive strength of this FGD gypsum-based mortar mainly depends on clusters of ettringite.

## 1. Introduction

The main chemical composition of gypsum (dehydrate) is sodium sulphate (CaSO_4_) which can be dehydrated to bassanite (hemihydrate) and anhydrite (anhydrous). Its elastic modulus, splitting tensile strength, fracture toughness, and flexural strength are highly correlated to its porosity [1,2]. FGD gypsum is a manmade gypsum from the flue gas desulfurization (FGD) process of air pollution controls at coal-fired power factories. This by-product has almost the same composition as natural gypsum [3,4,5]. Resource recovery of the FGD process has been emphasized for many years, especially for FGD gypsum [6]. FGD gypsum has been made into prefabricated building elements such as bricks, blocks, and wallboards [7], but some mercury is likely to be embedded in FGD gypsum due to the wet FGD scrubber [8]. FGD gypsum also has been used in Portland cement [9], calcium aluminate cement [10], calcium-sulfate-bearing material [11], and various binders of blended cement [12,13]. Hybrid FGD gypsum-based materials blended with textile fiber wastes or styrene-acrylic emulsion have already been made [14,15].

Furthermore, FGD gypsum could be utilized as raw materials of α-calcium sulfate hemihydrate and anhydrite [16,17,18]. The flexural strength of plasters made by FGD gypsum is higher than those made by gypsum due to the short setting time and more needle-shaped crystals [19]. Together with ground granulated blast furnace slag (GGBS) in concrete, FGD gypsum, after thermal pretreatment, can promote the growth of ettringite [20]. Not treated as an additive, FGD gypsum is made into a binder which contains GGBS, cement, lime powder and water glass [5]. Therefore, it would be possible to take full advantage of FGD gypsum and GGBS by making one new gypsum-based binder based upon super-sulfated cement (SSC).

The typical compositions of SSC are GGBS (75~85 wt.%), a sulphate composition like gypsum (10~20 wt.%), and an alkaline activator (less than 5 wt.%) like ordinary Portland cement (OPC) to accelerate corrosion of the glass network of GGBS. The compressive strength of SSC mostly depends on the formation of ettringite, so GGBS with Al_2_O_3_ content (higher than 13~15 wt.%) is strongly recommended [21]. There is an investigation which tries to use low-reactivity GGBS, where results show that the early compressive strength of SSC is not increased, and there is only a slow increase later [22]. SSC made with GGBS of low Al_2_O_3_ content (7 wt.%) presents a lower degree of hydration and less amounts of hydrates (ettringite and C-S-H), which results in a higher porosity and inferior mechanical properties [23]. Both [22,23] have suggested that the keystone for SSC made with GGBS of low Al_2_O_3_ content is the dissolution acceleration of the glass network of GGBS. Low calcium Class F fly ash may be an effective alumina supplier for SSC [24]. The alkaline activator in SSC can be OPC in [21,23], KOH in [22], anhydrite in [25], clinkers in [26,27]. Circulating fluidized bed combustion fly ash can be used as the alkaline activator which contains anhydrite and portlandite [24].

The remarkable advantages of SSC are the low hydration heat [26] and the superior resistance to sulfate attacks [28]. A series of investigations have been explored for the immobilization of low and intermediate level nuclear wastes using SSC [29,30]. To create wider applications for by-products, phosphogypsum is used for SSC [27]. A modified SSC has been made by [31], which is composed of sodium silicate activated slag, gypsum, and cement. Only ladle slag and gypsum could be made into a by-product binder [32]. Thus, one new binder is feasible, which mainly contains FGD gypsum and GGBS, activated by OPC according to the mechanism of SSC.

Here, one new binder-based mortar is made which partly originates from SSC but contains more FGD gypsum. Compressive strength and durability performances of this FGD gypsum-based mortar under different curing conditions are studied based on its microstructure evolutions, which provide an alternative method to reuse FGD gypsum to make new sustainable binders. We present the pioneering experiments of applications of ^1^H nuclear magnetic resonance (NMR) on the porosity of gypsum-based binders to motivate further NMR investigations on the porosity of cementitious materials.

## 2. Materials and Methods

### 2.1. Materials

**Figure 1:**
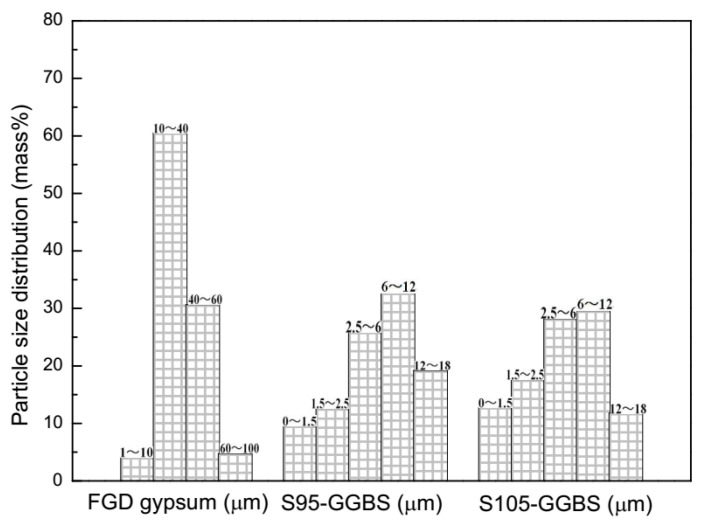
The particle size distributions of FGD gypsum and GGBS.

FGD gypsum, S95-GGBS, and S105-GGBS were provided by Shanghai Baotian New Building Materials Co., Ltd (Shanghai, China). The chemical compositions of these raw materials were listed in Table 1. The physical state of the FGD gypsum was a powder-like condition in a dark brown color, and had 15.6 wt.% free water, which was dried in a vacuum oven at 40 °C for 3 days. The OPC (P.O.52.5 cement, Conch Cement Corp., Wuhu, China) was kept in the commercial state, with a density of 3200 kg/m^3^, the specific surface was 352 m^2^/kg, and the initial setting time and the final setting time were 142 min and 275 min. The compressive strength of the OPC in the commercial state was 38.7 MPa and 61.2 MPa at 3 days and 28 days, respectively. The flexural strength of the OPC in the commercial state was 6.9 MPa and 8.1 MPa at 3 days and 28 days, respectively. S105-GGBS shared the same composition as S95-GGBS, only it was finer in particle size distribution. The average particle diameters of FGD gypsum, S95-GGBS, and S105-GGBS were 35.59 μm, 10.36 μm, and 7.74 μm, respectively. Detailed particle size distributions are demonstrated in Figure 1. It can be seen from Figure 1 that the particle size distributions of the FGD gypsum were mainly distributed from 10 μm to 40 μm (61 wt.%) and from 40 μm to 60 μm (32 wt.%). Comparatively, the particle size distribution of S95-GGBS ranged from 6 μm to 12 μm (33 wt.%) and from 2.5 μm to 6 μm (26 wt.%), which were obviously much finer than the S105-GGBS. CaCl_2_ and Ca(OH)_2,_ as alkaline activators, were purchased from Shanghai Sinopharm Group Chemical Reagent Co., Ltd (Shanghai, China). The dosages of the two activators were 1–3 wt.%. Ordinary river sand was used as the aggregate, with a fineness modulus of 2.4 and a bulk density of 1450 kg/m^3^. Tap water was used for mixing samples.

### 2.2. Compressive Strength

Mortars (40 × 40 × 160 mm^3^) were made and tested according to Chinese Standard-GB/T 17671-1999 [33]. The procedure was performed at a loading rate of (2400 ± 200) N/s. Each result was the average value of five samples. The water-to-cement ratio of the mortars was 0.5, the sand-to-cement ratio of the mortars was 1/3, with the detailed proportions listed in Table 2. Samples were cured under normal curing conditions of 20 ± 1 °C and (65 ± 1%) Relative Humidity (RH).

The effects of S105-GGBS on the compressive strength of the FG-4550 mortars were investigated under normal curing conditions. S95-GGBS was replaced by S105-GGBS at 15 wt.%, 30 wt.%, 45 wt.%, 60 wt.%, 75 wt.% and 100 wt.%, with the detailed proportions listed in Table 3.

Steam curing conditions were applied to the FG-4550 samples. The brief process was as follows. After standing for 2 h at room temperature, fresh samples with molds were cured in the steam chamber for 8 h at 60 °C, with a heating rate of 20 °C/h. Then they were demolded and cured under normal curing conditions. The effects of CaCl_2_ and Ca(OH)_2_ on the compressive strength of FG-4550 mortars under steam curing conditions were compared.

### 2.3. Resistance to Water

FG-4550 mortars first were cured under steam curing conditions and then cured under normal curing conditions for 28 days. Resistance to water was evaluated by the compressive strength of the mortars after immersion for 7, 28, 56, and 90 days. The temperature of the water immersion process was 20 ± 1 °C and samples were placed on a plastic frame located in the middle of the water in a stainless steel container. Water in the stainless steel container was changed every 7 days until the end.

### 2.4. Resistance to Carbonation

Tests of resistance to carbonation of FG-4550 mortars followed Chinese Standard-GBT50082-2009 (the acceleration carbonation) [34]. Following steam curing conditions, samples were cured for 26 days under normal curing conditions, then dried for 48 h in an oven at 60 °C. Samples were taken into the carbonation chamber after being treated with paraffin. The condition of the carbonation chamber had CO_2_ concentrations at (20 ± 3)%, temperature at (20 ± 2) °C, and relative humidity at (70 ± 5)%. Carbonation depths were gained by spraying with phenolphthalein solutions (0.1 mol/L).

### 2.5. Resistance to Freeze-Thaw Cycles

Resistance to freeze-thaw cycles of FG-4550 mortars were evaluated after 30 freeze-thaw cycles. Prior to the freeze-thaw cycles, the FG-4550 mortars were cured under steam curing conditions, then under normal conditions for 28 days. The procedure of one freeze-thaw cycle was: (1) immersion in water at 15–20 °C for 2 days; (2) the weight-recording after being taken from the water and wiped dry; (3) the freeze-thaw cycle which began at (−25 ± 2) °C for 4 h; (4) the removal step after timing and warming in water at 15–20 °C for 4 h.

### 2.6. Drying Shrinkage

The shrinkage ratios of FG-4550 mortars were assessed using Chinese Standard-JC/T603-2004, which was used for another FGD gypsum-based material [5]. FG-4550 mortars first were under steam curing conditions and then cured under normal conditions for 7 days. Length changes of the FG-4550 mortars were observed from 7 days to 14, 21, 28, 56, 90 days. Shrinkage ratios were calculated using Equation (1), in which L_0_ was the original length of the sample, L_t_ was the length of the sample at period t (day), and ∆L was the shrinkage ratio.

∆L (%) = L_0_/L_t_ × 100% (1)

### 2.7. Microstructure Analysis

FG-4550 pastes (20 mm × 20 mm × 20 mm) were made at w/c = 0.5. The mineralogy of the FG-4550 pastes at 3 days and 28 days under normal curing conditions and steam curing conditions were tested using X-ray diffraction (XRD) equipment (Bruker, Karlsruhe, Germany) of Rigaku-D/max2550VB3+, from 5° to 75° at 5°/min with a Cu Kα radiation. XRD patterns were presented using the software Jade 6.5. (version 6.5, MDI, Livermore, CA, USA). Scanning electron microscopy (SEM) of FG-4550 mortars were demonstrated by FEI Quanta FEG 200 (FEI, Hillsboro, OR, USA). Prior to the microscopic observations, samples were dried in a vacuum drying oven at 40 °C for 72 h, then crushed and coated with gold for the test with an accelerating voltage of 15 kV and a current of 20 mA.

^1^H Nuclear magnetic resonance (NMR) has been successfully used to investigate the morphology of C–S–H [35,36], carbonation fronts in cement paste [37], the porosity of lime-pozzolan mixtures and mortars [38,39], and superplasticizers in fresh cement [40,41]. One method used by [37], ignored fast diffusion. The NMR transverse relaxation rate of pore water in a hydrated cement paste could be simplified using Equation (2) as cited by [42]. Using Equation (2), 1/T_2_ was the relaxation rate, $\rho_{2}$ was the surface relaxivity, and S/V was the surface-to-volume ratio of the pore space. The surface relaxivity was dependent upon the chemical composition of the solid interacting with pore water molecules [37].

(2) $$\frac{1}{T_{2}}=\rho_{2}\frac{S}{V}$$

During this experiment, *T_2_* NMR relaxation times were measured using the method of [37]. Related cement pastes (20 mm × 20 mm × 20 mm) were made at w/c = 0.5 and cured under different curing conditions. Then, they were cured under water at 20 ± 1 °C. Prior to the NMR tests, samples were broken into pieces and immersed in alcohol to stop cement hydration for 2 days. The NMR tests were performed using a PQ-001 NMR (Niumag Corporation, Shanghai, China) as done in previous investigations in our laboratory [38,39]. The proton resonance frequency was 23 MHz and the permanent magnet was kept at 32 °C. The transverse magnetization decay was measured with the Carr-Purcell-Meiboom-Gill (CPMG) technique. The CPMG measurement parameters were echo time = 100 μs, number of echoes = 500, and number of scans = 128.

## 3. Results and Discussion

### 3.1. Compressive Strength under the Normal Curing Condition

**Figure 2:**
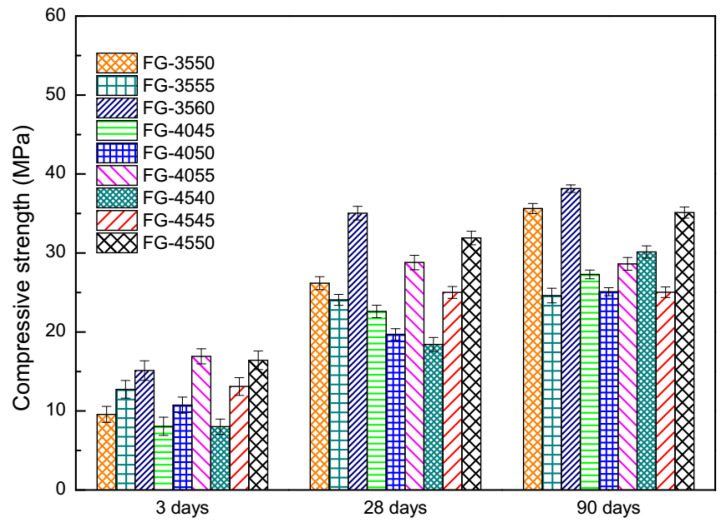
Compressive strength of FGD gypsum-based mortars with S95-GGBS under normal curing conditions.

The compressive strength of FGD gypsum-based mortars with S95-GGBS under normal curing conditions are demonstrated in Figure 2. Occurring at 3 days, on the dosage of 35 wt.% FGD gypsum, FGD gypsum-based mortars blended with the maximum GGBS (60 wt.%) showed the highest compressive strength (FG-3560). The same results were found using FGD gypsum-based mortars under the condition of 40 wt.% FGD gypsum (FG-4055) and the condition of 45 wt.% FGD gypsum (FG-4550). Among FGD gypsum-based mortars on three dosages of FGD gypsum (35–45 wt.%), the compressive strength of FG-4055 occupied the top position. All of the lowest compressive strength values are found in FGD gypsum-based mortars with the minimum GGBS (FG-3550, FG-4045, FG-4540) for each dosage of FGD gypsum (35 wt.%, 40 wt.%, 45 wt.%). The compressive strength of FG-3550 was stronger than that of FG-4045 and FG-4540.

Occurring at 28 days, both the compressive strength of FG-3560 and the compressive strength of FG-3550 experienced considerable changes. The increase on the compressive strength of FG-3560 was from 15.2 MPa (3 days) to 35.1 MPa (28 days), which was the highest compressive strength. The increase of the compressive strength of FG-3550 was from 9.6 MPa (3 days) to 26.3 MPa (28 days). The second and third rankings regarding the compressive strength of FGD gypsum-based mortars went to FG-4550 and FG-4055, respectively. Similar to the situation at the age of 3 days, the lowest compressive strength went to FG-4540.

Occurring at 90 days, the compressive strength of FG-3560 was still the highest, but the second position was taken by FG-3550 (35.6 MPa), which was a little higher than FG-4550 (35.2 MPa). The compressive strength of FG-4540 (30.5 MPa) took fourth position, showing different situations at 3 days and 28 days. FG-3555 (24.6 MPa), FG-4050 (25.3 MPa) and FG-4545 (25.6 MPa) could share the lowest compressive strength due to the small measurement gaps between them.

According to the hydration scheme of ordinary SSC [21], the amount of hydrates, or the origin of compressive strength, highly depends on the quantity of GGBS and the degree of Al^3+^ ions released from the GGBS. Thus, the lower compressive strength was reasonably found in FGD gypsum-based mortars with less GGBS. The increase in the compressive strength of FG-3550 after longer curing times also can be understood using the mechanism of SSC [21]. The compressive strength of SSC is mainly represented by ettringite, which is generated during early hydration. The compressive strength of ordinary Portland cement (OPC) is mainly represented by C–S–H, which keeps growing during the whole hydration time. It could be concluded, based on the mix proportion of FG-3550, that the compressive strength of FG-3550 depended on both ettringite and C–S–H.

**Figure 3:**
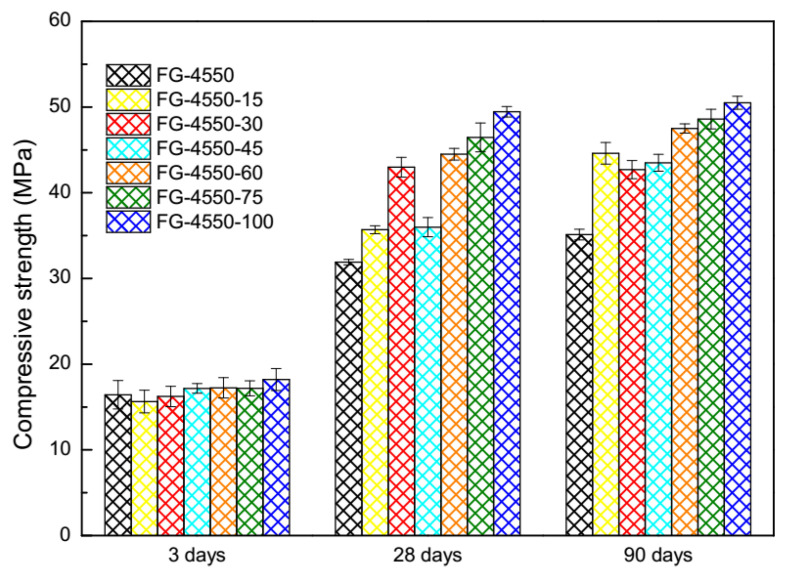
Compressive strength of FGD gypsum-based mortars replaced by S105-GGBS under normal curing conditions.

Concerning the mix proportion of FG-4550 with the highest amount of FGD gypsum, the influence of the fineness of GGBS on the compressive strength was tried on mortars of this selected mix proportion through replacing S95-GGBS with S105-GGBS, as seen in Figure 3. Occurring at 3 days, S105-GGBS showed little effect on the development of the compressive strength. The compressive strength of FG-4550-100 (the total replacement) just surpassed that of FG-4550. During the curing times, prolonged to 90 days, all the compressive strength of FGD gypsum-based mortars with S105-GGBS showed tremendous progress. The orders of the compressive strength of these mortars were regularly followed with dosages of S105-GGBS.

### 3.2. Compressive Strength of Activated FG-4550 under the Steam Curing Condition

**Figure 4:**
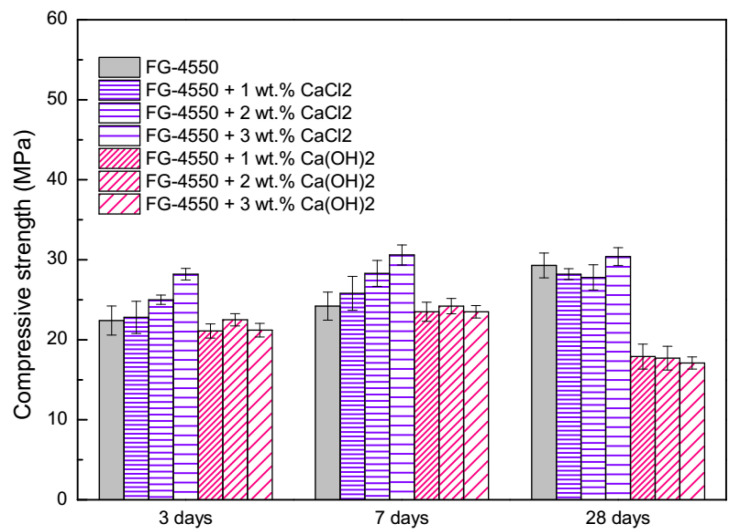
Compressive strength of activated FG-4550 under steam curing conditions.

The compressive strength of FG-4550 blended with different chemical activators under steam curing conditions is illustrated in Figure 4. It can be seen that increases in CaCl_2_ on the compressive strength of FG-4550 are considerable, meanwhile the reductions in Ca(OH)_2_ on the compressive strength of FG-4550 are obvious. Whatever the curing times, the biggest compressive strength was for FG-4550 blended with 3 wt.% CaCl_2_. The orders of compressive strength of activated FG-4550 were regularly followed by dosages of CaCl_2_ at the age of 3 days, 7 days, and 28 days. During the 3–28 days, the compressive strength of FG-4550 without activators increased gradually, from 79.5% to 96.5% of the FG-4550 blended with 3 wt.% CaCl_2_. All the compressive strengths of FG-4550 blended with Ca(OH)_2_ showed remarkable downsides during the whole curing time. Occurring at 28 days, the compressive strength of FG-4550 blended with 1 wt.%, 2 wt.%, 3 wt.% Ca(OH)_2_ was only 58.9%, 58.2%, and 56.3% of the FG-4550 blended with 3 wt.% CaCl_2_, respectively. Comparatively, the compressive strength of FG-4550 under steam curing conditions at 3 days (22.4 MPa in Figure 4) was higher than when under normal curing conditions (16.4 MPa in Figure 2), but lower at 28 days (29.3 MPa and 31.9 MPa, respectively). It seems that the steam curing condition might not be helpful for the development of the compressive strength of FG-4550.

Different to diethanol-isopropanolamine (DEIPA) accelerating the hydration process of the aluminate phases [43], it is well known that CaCl_2_ mainly accelerates the hydration process and the development of the compressive strength of tricalcium silicate (C_3_S) [44], which leads to a better compressive strength of FG-4550. Given that there was only 5 wt.% cement in the raw materials, contributions of the steam curing condition to the compressive strength by microstructure modifications [45] might be negligible, as demonstrated in Figure 4. Based on the mechanism of SSC [21], the negative effects of Ca(OH)_2_ on the compressive strength of FG-4550 could be attributed to interference by Ca(OH)_2_ on the original alkaline activator in the hydration system.

### 3.3. Durability of FG-4550 under the Steam Curing Condition

**Figure 5:**
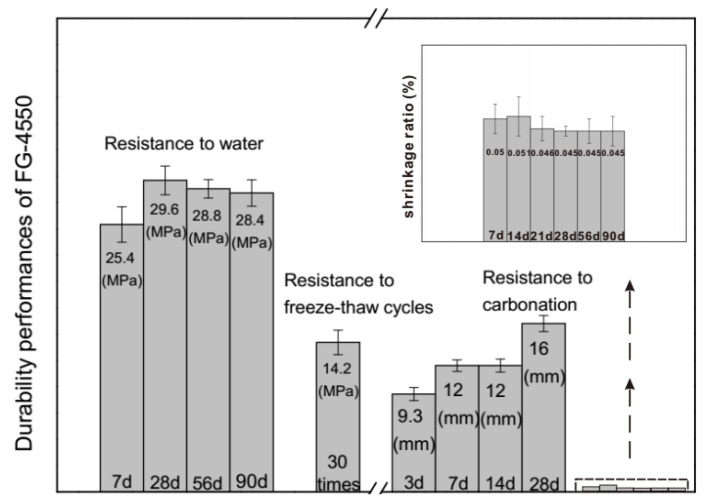
Durability performances of FG-4550 under steam curing conditions.

Durability performances of FG-4550 are demonstrated in Figure 5. During the immersion times between 7–90 days, the increasing amplitude of the compressive strength of FG-4550 became smaller after 28 days and remained stable from 56 days to 90 days. Since the main reason for the deterioration of gypsum-based materials is crystallizing with water, whatever gypsum, or bassanite (hemihydrate), or anhydrite (anhydrous) used, the stability of the compressive strength of FG-4550 under water means the compacted microstructure originated from the dense porosity and full hydration of GGBS. The compressive strength of FG-4550 after freeze-thaw cycles (30 times) was only 14.2 MPa, which revealed the poor behaviors of this FGD gypsum binder at a low temperature environment. Compared to the superior performance of the FGD gypsum binder after 200 freeze-thaw cycles (34.57 MPa) [5] and the typical compositions of SSC [21], it could be assumed that the extra FGD gypsum in FG-4550 might play a negative role in its resistance to freeze-thaw cycles. Gypsum is composed of needle-like gypsum crystals which have accumulated pores as a permeable network system inside matrix [46,47] as well as a carbonation front (or carbonation depth) correlated to porosity [37]. The stabilization of the carbonation depth at 7 days and 14 days represented that the stable porosity of FG-4550 during that period, while the augmentation of the carbonation depth from 3 days to 7 days and from 14 days to 28 days revealed critical evolutions at these days. The shrinkage ratio of FG-4550 was kept between 0.045% and 0.051% from 7 days to 90 days. According to the shrinkage ratios of cement-based materials, the volume shrinkage depends on the physical shrinkage from the evaporation of free water and the chemical shrinkage from the hydration process [5]. Due to the steam curing conditions, the hydration of GGBS was accelerated and there was almost no free water inside FG-4550, which led to the low shrinkage ratios shown in Figure 5.

### 3.4. Microscopic Characteristics of FG-4550

**Figure 6:**
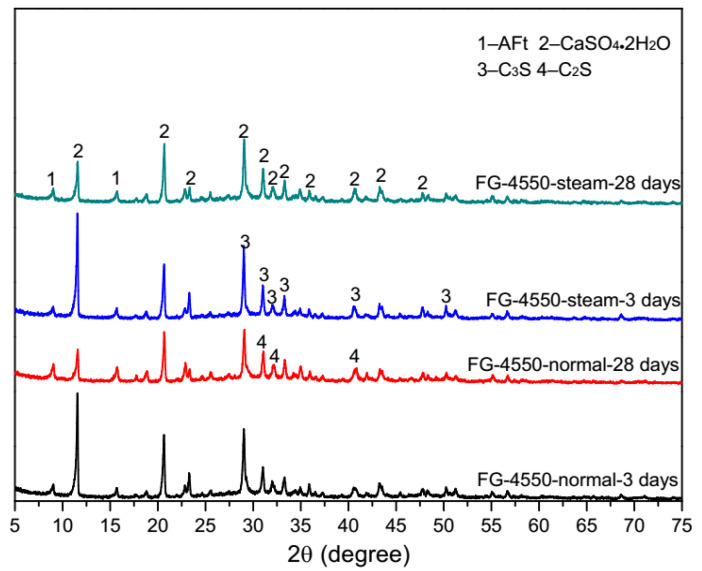
XRD patterns of FG-4550 pastes under steam curing conditions and normal curing conditions.

XRD patterns of FG-4550 pastes under different curing conditions are shown in Figure 6. Under normal curing conditions, the intensity of gypsum was considerably decreased from 3 days to 28 days. Meanwhile, the intensity of tricalcium (C_3_S) and the intensity of dicalcium (C_2_S) were seemingly unchanged. The intensity of ettringite (AFt) was obvious at 3 days and 28 days, which means that the hydration process of SSC happened. Found in the raw materials of FG-4550, there were almost 20 wt.% gypsum more than in the raw materials of SSC described by [20]. Furthermore, there was nearly 20 wt.% less GGBS in FG-4550 to trigger the full hydration process of SSC. Consequently, the alkaline activators in FG-4550 might not have been exhausted so the clinker phases in the cement still were seen at 28 days, especially tricalcium silicate (C_3_S). Under steam curing conditions, the situations of the mineralogy were quite similar, which means that the steam curing condition could be an alternative for FGD gypsum-based mortars to shorten the manufacturing schedule at job sites.

**Figure 7:**
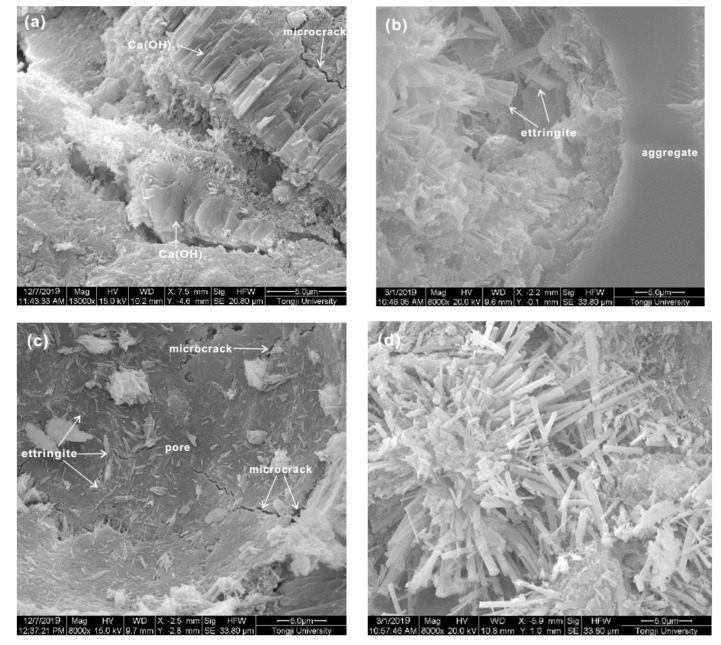
SEM images of FG-4550 mortars under different curing conditions. (**a**,**b**) FG-4550 at 3 days and 28 days under normal curing conditions; (**c**,**d**) FG-4550 at 3 days and 28 days under steam curing conditions.

SEM images of FG-4550 mortars under different curing conditions are shown in Figure 7. Stack layers of Ca(OH)_2_ at 3 days revealed that cement hydration started in the extra cement grains, which is in accordance with the XRD patterns of FG-4550 in Figure 6. The rod-like ettringite (AFt) was found near to aggregates at 28 days, as shown in Figure 7b, and the clusters of rod-like ettringite (AFt) also were observed under the steam curing conditions, as shown in Figure 7d. These overlapped crystals are highly responsible for the compressive strength of FG-4550. There are a few microcracks shown in Figure 7a,c, which might do harm to the compressive strength of FG-4550 at 3 days.

**Figure 8:**
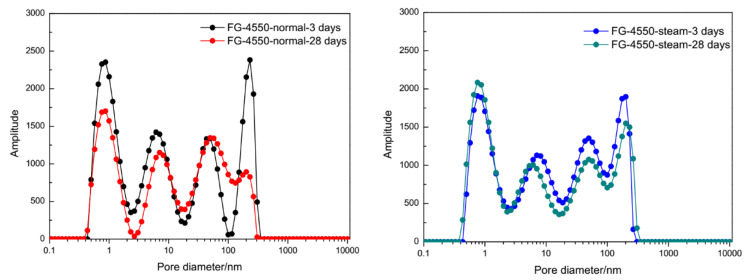
Pore diameter distributions of FG-4550 pastes under different curing conditions inverted by *T_2_* relaxation time of NMR.

Pore diameter distributions of FG-4550 pastes inverted by T_2_ relaxation time are shown in Figure 8. Under normal curing conditions, the peaks in the curve of amplitude were discrete at 3 days which means a high porosity. The peaks in the curve of amplitude were mild at 28 days which means a finer porosity. Under steam curing conditions, the peaks in the curve of amplitude at 3 days were similar to those at 28 days. This situation implies that the development of porosity in FG-4550 pastes was nearly stopped after the steam curing process. The obvious evolution of porosity in FG-4550 pastes under normal curing conditions was owing to the filler effect of developing hydrates from 3 days to 28 days. The slight evolution of porosity in FG-4550 pastes under steam curing conditions might be owing to the developed hydrates in the steam chamber for 8 h at 60 °C. The filler effect of hydrates for FG-4550 are mostly referred to the rod-like ettringite (AFt) in Figure 7b,d.

## 4. Conclusions

This article aimed at investigating the compressive strength and durability of one FGD gypsum-based mortar. Variations of raw materials, curing conditions, and chemical activators on the compressive strength were considered. Durability performances of this FGD gypsum-based mortar under one selected mix proportion (FG-4550, with the most FGD gypsum at 45 wt.%) under steam curing conditions for resistance to water, resistance to freeze-thaw cycles, and drying shrinkage were evaluated.

The compressive strength of this FGD gypsum-based mortar under normal curing conditions could be strengthened with the increase of GGBS (as much as 60 wt.%). Improving the fineness of GGBS was also an effective method for the development of compressive strength. The activated effect of CaCl_2_ was better than that of Ca(OH)_2_ on the compressive strength of FG-4550 under steam curing conditions. The stable performances of resistance to water, resistance to carbonation, and the shrinkage ratio of FG-4550 owed to the modified porosity under steam curing conditions. The resistance to freeze-thaw cycles of FG-4550 was inferior, which was partly due to the extra FGD gypsum in the raw materials. Entangled ettringite was the main hydrate of this FGD gypsum-based binder.

## Figures and Tables

**Table 1:** Chemical compositions of raw materials (wt.%).

Components	SiO_2_	CaO	Al_2_O_3_	Fe_2_O_3_	MgO	Na_2_O	K_2_O	TiO_2_	SO_3_	Crystal Water
FGD gypsum	1.42	32.2	2.17	0.75	0.52	0.08	0.1	0.1	41.25	18.28
Cement	20.8	61.3	6.34	3.1	1.0	0.35	0.5	0.29	2.3	-
S95-GGBS	31.2	41.4	14.6	0.4	0.3	0.28	0.27	0.66	2.3	-

**Table 2:** Mix proportion of mortar samples (wt.%).

Sample	FGD Gypsum	S95-GGBS	Cement
FG-3550	35	50	15
FG-3555	35	55	10
FG-3560	35	60	5
FG-4045	40	45	15
FG-4050	40	50	10
FG-4055	40	55	5
FG-4540	45	40	15
FG-4545	45	45	10
FG-4550	45	50	5

**Table 3:** Mix proportion of FG-4550 replaced by S105-GGBS (wt.%).

Sample	FGD Gypsum	Cement	S95-GGBS	S105-GGBS
FG-4550	45	5	50	0
FG-4550-15	45	5	42.5	7.5
FG-4550-30	45	5	15	15
FG-4550-45	45	5	27.5	22.5
FG-4550-60	45	5	20	30
FG-4550-75	45	5	12.5	37.5
FG-4550-100	45	5	0	50
